# HK-OxVPS: an adaptation of the Oxford Visual Perception Screen for the Cantonese speaking population in Hong Kong

**DOI:** 10.3389/fneur.2025.1632814

**Published:** 2025-10-27

**Authors:** Tsz Ying Flora Loh, Kathleen Vancleef

**Affiliations:** Department of Psychology, Durham University, Durham, United Kingdom

**Keywords:** perception, visual impairment, cognitive impairment, assessment, normative data, translation, Hong Kong, Cantonese

## Abstract

The Oxford Visual Perception Screen (OxVPS) is a screening tool recently developed for visual perception deficits that occur after a stroke, such as difficulties in recognizing objects, faces, and reading. The OxVPS allows for quick and accessible screening through 10 subtests, including naming pictures and matching shapes. Hong Kong has many stroke survivors, but only the wealthy can afford comprehensive cognitive assessments, highlighting the need for affordable screening tools in public healthcare. This study developed the Hong Kong—Oxford Visual Perception Screening test (HK-OxVPS), a translation and cultural adaptation of the OxVPS. Normative data from the Hong Kong neurologically healthy population was collected, and cut-off scores for each subtest were derived from the distribution of scores from 95 native Cantonese-speaking participants (50–95 years old). Comparison of cut-off scores with the cut-off scores of the UK version of the OxVPS found a general trend for lower scores on the HK-OxVPS, even on non-linguistic and non-culturally relevant subtests (intercept in Delta plot analysis = 5.69). Age, education, and visual acuity were not significant influencers of HK-OxVPS test performance (*p*-values 0.96, 0.16, and 0.07, respectively). However, qualitative inspection of patterns in the data of participants who were unable to complete specific subtests suggested a relationship between age and education on subtest completion. Further validity and reliability testing, as well as improvements to increase test completion, may be necessary to ensure suitability for use with Cantonese speaking stroke survivors.

## Introduction

Visual perception is the process by which our brain interprets and comprehends visual stimuli. Light entering our eyes is converted into electrical signals by our retina and is then transferred via the optic nerves to the visual cortex. At that stage, which we refer to as visual perception, meaningful representations of what we see, can be generated (1). A brain injury like a stroke can lead to visual perception deficits that make it difficult for an individual to structure and interpret visual information. Visual perception deficits can exhibit difficulty organizing, processing, interpreting, and responding appropriately to visual information. They can manifest as the inability to recognize familiar faces (prosopagnosia), the inability to perceive and interpret shapes (apperceptive agnosia) or the inability to acknowledge part of the visual field (visual neglect) (2).

Visual perception deficits can impact an individual's physical health, mental health, and independence. Returning to work, driving, and in severe cases, daily life activities may be more challenging, requiring assistance, thus increasing dependence (2–4). Visual perception deficits can hinder an individual's everyday functioning and leave them feeling helpless and worthless (2, 5). The severe impact of visual perception deficits on a patient's everyday life highlights the need for thorough screening and effective rehabilitation.

Despite its high prevalence with estimates up to 76% (6–9), visual perception impairments after acquired brain injury are rarely identified. This is potentially due to limited education for professionals on visual perceptual deficits and the lack of a standardized screening test (10, 11). Existing assessment tools such as the Behavioral Inattention Test (12) or the Hooper Visual Organization Test (13) only cover a partial aspect of visual perception and therefore fail to provide an encompassing view of a stroke survivor's visual perception abilities. Moreover, tests like the Rivermead Perceptual Assessment Battery (14), the Visual Object and Space Perception test [VOSP, (15)], and the Birmingham Object Recognition Battery [BORB (16)] are impractical for stroke patients due to their lengthy testing time, complexity, and the need for extensive staff training (17, 18). As a result, many health professionals depend on patients to provide information about their vision. Self-reporting methods can be often unreliable due to their subjective nature and lead to an under-identification (2, 11). Such under-identification is detrimental as screening is essential in providing early interventions and accommodations for stroke survivors (19).

According to orthoptists and occupational therapists, who are most often responsible for visual perception screening, an ideal screening test for visual perception deficits should be quick and adaptable to a stroke survivor's condition, require minimal training for administrators, and be accessible to patients with expressive aphasia following a stroke (11). The Oxford Visual Perception Screening Test (OxVPS) attempts to address the need for such a post-stroke visual perception screening test (20). The OxVPS comprises of 10 subtests that can screen for a multitude of visual perception disorders. Tasks include counting stars presented on a page, replicating a geometrical figure by drawing and naming simple line drawings of objects, etc.

The OxVPS was developed in the United Kingdom and it is currently only available in British English. Cut-off scores are based on a sample of neurologically healthy people living in the United Kingdom (20). Although the sample is representative in age for a United Kingdom population of stroke survivors, the authors acknowledge the lack of diversity in ethnicity and education in their sample and the potential effect on (cut-off) scores. It is therefore unclear whether OxVPS is appropriate to use with ethnic minorities, people with a low education and in populations outside the United Kingdom is therefore unclear.

Accessible reliable medical screening technologies are an important problem in global healthcare. The phrase “trickle-down science” refers to the misconception that the concentration of resources and opportunities in science from developed countries will produce the “best science” and subsequently “trickle-down” and benefit those of less developed countries as well (21). This myth of “trickle-down science” and the over-reliance on the West further exasperates the barrier to reliable medical screening technologies as Western healthcare solutions may not apply to local problems or local problems or contexts of the East (21). For neuropsychological tests, the two significant obstacles are culturally inappropriate content and language barriers (22). In the domain of visual perception, cultural differences have been observed in color naming (23), perception of facial expressions (24), and scene perception (25). This is reflected in differences in performance on visual neuropsychological tests (26) and in eye movement scanning patterns between a British and an Indian population (27). Although, differences are less profound when comparing performances between different Western European cultures (28, 50). The language barrier can be especially problematic for countries with different languages or distinct local dialects and for immigrant populations. As a result, there is an urgent need for translated versions of medical screening exams like the OxVPS.

As the fourth most common cause of death, third most common cause of hospital admission and the most common condition for long-term residential care, strokes and any resulting disability affect the lives of many in Hong Kong (29). Hong Kong also needs more education regarding stroke and visual perceptual impairments and a consensus on what they are (30). A significant disparity exists between the quality of private healthcare that the rich can afford and accessible public healthcare (31). The accessibility and inexpensive nature of the OxVPS can open access to visual perception screening through the public healthcare system. A Hong Kong Cantonese translation of the OxVPS can address this need for a post-stroke visual perception screening test. Furthermore, it has the potential to reduce language barriers of neuropsychological testing across East Asia, and in immigrant communities worldwide, promoting more accurate screening and healthcare. Although, a different cultural context might make direct application challenging.

This study sets out to translate and develop a culturally relevant and meaningful adaption of the OxVPS into traditional Chinese for Cantonese usage, known as the Hong Kong—Oxford Visual Perception Screen (HK-OxVPS) followed by collection of normative data from the middle to old age population in Hong Kong. This data will help establish the typical range of performance for the HK-OxVPS and enable comparison with the original British version of the test.

## Materials and methods

### Development of HK-OxVPS

The OxVPS was translated into Chinese (Traditional) and adapted for use with Cantonese-speaking individuals, creating the Hong Kong—Oxford Visual Perception Screen (HK-OxVPS). Traditional Chinese is the writing system primarily used in Hong Kong, and Cantonese is Hong Kong's primary spoken language.

### Translation process

All English text within the OxVPS stimulus book and the examiner form were reviewed and translated following the International Test Commission Guidelines for Translating and Adapting Tests (22). Permission was obtained from the OxVPS copyright holders [conform Guideline 1 in (22)]. Forward translation was completed independently by two translators who were native Cantonese speakers and proficient in written Chinese and English [conform Guideline 6 in (22)]. One of the translators was the first author who had a background in Psychology and Cognitive Neuroscience. This translator was familiar with the test content, constructs, and testing principles [conform Guideline 4 in (22)]. Both forward translations were compared and reviewed. Disagreements were discussed, resolved, and a translation was accepted if a consensus was reached. The reconciled version was back-translated into English independently by a third translator proficient in both written Chinese and English who was naive to the original English-OxVPS. The back translation was then put against the original British-OxVPS. Further changes were made to ensure that the original meaning and test perspective were maintained from the translation [conform Guideline 6 in (22)].

### Cultural adaptation

The OxVPS was evaluated for construct validity in the Hong Kong population through review by two Hong Kong Clinical Psychologists familiar with the target population and clinical context. They reviewed both the overall screening test for correspondence with the concept of “visual perception” in Hong Kong and each of the subtests for equivalence in each of the symptoms the test screens for (e.g., associative agnosia, alexia, prosopagnosia) in a Hong Kong context [conform Guideline 1 in (22)]. Images and words used in the original OxVPS were reviewed and revised to be familiar to a Cantonese-speaking audience from Hong Kong by the first author, who is native to Hong Kong and has been a resident for 18 years [conform Guideline 5 in (22)]. Images and answer options that were not suitable were replaced following the same test principles and style as the original version. Changes were made with input from the developer of the original OxVPS and all translators involved to ensure the test instructions and items had a similar meaning to the original version [confirm Guideline 6 in (22)].

In the picture naming task, three images were considered culturally unfamiliar: a ladybug, swimming goggles, and a packing box. This was because either the corresponding Chinese written terms were deemed challenging, or the items were not part of daily life. Images of a cow, a mushroom, and a balloon replaced them. The choice of answer options for the new images followed the same principles as in the original version of OxVPS, where one of the answer options is the correct word describing the picture, one is a word visually related to the correct answer, one is a word semantically related, one is a word semantically and visually related, and one is a completely unrelated word.

Two images in the semantic information task, the tractor tire and pencil sharpener, were replaced by an image of a playground slide and rabbit. Here, the answer options followed the same principles as the original OxVPS, where the correct answer is the only word semantically related to the picture, and all the other words are semantically related to each other. For instance, the correct semantically related answer for the rabbit stimulus is “carrot” (hung lo baak 紅蘿蔔) while the incorrect answers were: “onion” (joeng cung 洋蔥), “banana” (hoeng ziu 香蕉), “tomato” (fan ke 番茄), and “potato” (syu zai 薯仔).

A culturally familiar story containing 130 characters replaced the original English reading subtest. Following the average reading speed of 259.5 ± 38.2 characters/min, this passage is meant to be read in about 30 s (32). The passage is about a grandchild having a Chinese tea meal (飲茶 yum cha) with their grandparents as they recount his recent school events. Exactly translating as “drink tea,” yum cha is a popular Cantonese custom where friends and family gather for brunch and share small dim-sum and other Cantonese dishes while drinking Chinese tea (33). Rather than making a purely semantic translation, the core concepts were preserved in the reading passage [conform Guideline 5 in (22)]. Following the same principles in the formation of morphemes and creating meaning by combining small units (34), the ten English compound words within the reading test were replaced with 31 Chinese words and expressions semantically reliant on each other. Duplicate characters and names were excluded.

**Figure 1:**
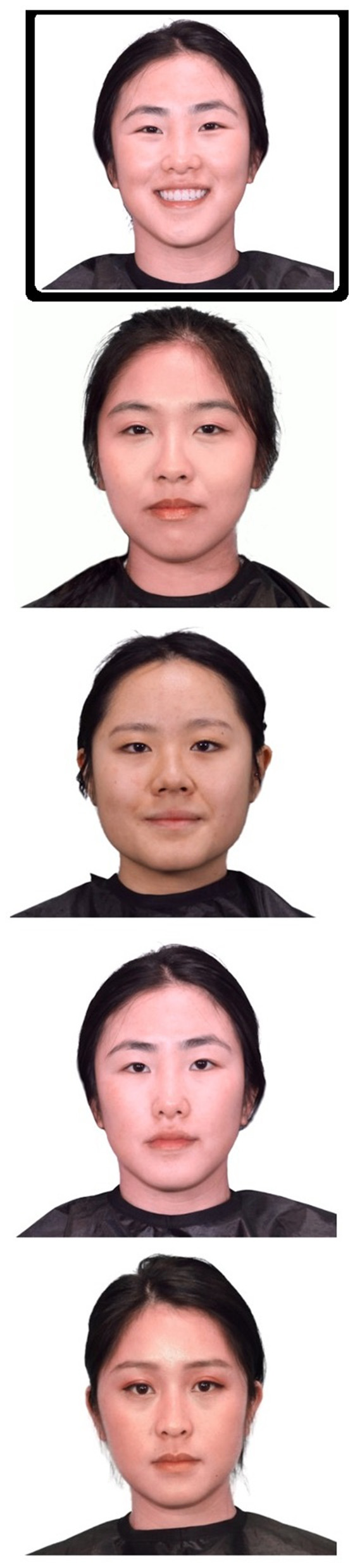
Example of facial recognition stimuli. Participants are presented with five faces and are asked to identify and match the target happy face on the top to one of the four neutral distractor faces below. © University of Durham.

To ensure accurate facial recognition abilities and to mitigate the presence of own-race bias (35), facial photographs used within the original OxVPS were replaced by Chinese ethnic facial photographs (Figure 1). Photographs of 37 Chinese ethnic volunteers (8 males and 29 females) were taken and edited to standardize size, background, and remove accessories like hair clips and jewellery. Participants were all students from Durham University and were recruited through convenience sampling, social media, and SONA, a participant recruitment system for Durham University Psychology students. This study was approved by the Durham University research ethics committee (PSYCH-2023-01-02T00_48_00-wvmw82) and in accordance with the ethical principles outlined in the Declaration of Helsinki (36). Informed consent was obtained prior to participation. Photographs of participants were grouped into sets of four based on similarities in appearances agreed by a panel of researchers. Each set of four resulted in one trial in which a photograph of a person was shown at the top of a page followed by images of four people with a different facial expression or from a different viewpoint. One of the bottom four photographs was of the same person as the target image at the top. A pilot study of the new face stimuli was carried out on Prolific (www.prolific.com), an online recruitment platform, with 29 participants from East Asian ethnicity, to establish which combination of emotional expressions and viewpoints give rise to an average performance of 80% correct answers. The combination that yielded 88% correctness (happy facial expression at the top, neutral facial expressions as answer options, all frontal views) was chosen as the optimal approach to minimize potential participant errors and biases while ensuring that the task would not be too difficult for middle- to old-aged participants.

### HK-OxVPS pilot

A convenience sample of 10 native Cantonese-speaking participants from Hong Kong took part in a pilot study to review the fluency and appropriateness of the HK-OxVPS [conform Guideline 8 in (22)]. Participants were between 45 and 75 years old; three were male, and seven were female. All participants were proficient in Cantonese, with no reported history of neurological conditions, speech and language impairments, or psychiatric conditions. Participants completed the HK-OxVPS (see Procedures and Materials below). Their quantitative scores on individual items were reviewed by the research team and the participants provided qualitative feedback on the fluency and cultural comprehension of the test through a brief post-test interview. They were asked to comment on the wording of the instructions, the response options, and to suggest alternative phrasing if appropriate. The pilot also allowed us to evaluate the appropriateness of the administration of the HK-OxVPS such as the paper-and-pencil format [conform Guideline 14 in (22)].

All participants were able to complete the test with ease and each participant made a maximum of three errors. Across participants 20% of items were answered incorrectly: most errors were made in the first six tasks; no errors were made in the reading task and the figure copy task; cancellation scores were within the normal range for all but one participant. The most common mistakes were in the face recognition task and the semantic information task. Four participants made a mistake in the facial recognition task and stated that they found the task difficult compared to the other tasks and required more careful thinking, though no further changes were made as their performance level was still above 80% and mistakes were not systematic. Five out of 10 participants answered one of the trials in the semantic information task incorrectly, answering “Hong-Kong styled diner” (茶餐廳 cha chaan teng) in response to stimuli of a cup instead of the correct answer, which was “drink” (飲 yum). This was most likely due to use of similarly styled cups commonly found in “Hong-Kong styled diners.” To avoid confusion, the answer option 茶餐廳 cha chaan teng was replaced with “straw” (飲管 yum goon). When asked about the fluency, comprehension and cultural appropriateness of the test, all participants answered that the test was straightforward and comprehensible. Image and response options used within the test were culturally familiar, with no participant left puzzled. Participants fed back that the test administration was appropriate and well-conducted. Four participants with professional experience in working with an elderly population gave further advice for improvement, such as guidance on tone of voice, speed of test administration, and body language.

### Normative data collection

The inclusion criteria for participants in the normative study were: no history of neurological conditions. Recruitment was stratified by age groups to match age ranges of stroke survivors as reported in Hong Kong's Stroke prevalence statistics [see Table 1; (29); conform Guideline 9 in (22)]. Participants were recruited through convenience sampling from public spaces, religious organizations, soup kitchens, and offices. They were compensated with vouchers, though 13 participants denied compensation.

**Table 1:** Age group matching between study participants and Hong Kong stroke prevalence.

**Age groups (years)**	**Percentage of Hong Kong stroke patients per age group**	**Recruitment targets**	**Number of study participants**
<45	0.62%	1	1
45–54	7.85%	7	9
55–59	9.09%	8	7
60–64	8.26%	7	15
65+	74.17%	67	63
Total	100%	90	95

This study was approved by the Durham University research ethics committee (PSYCH-2023-01-02T00_48_00-wvmw82) and in accordance with the ethical principles outlined in the Declaration of Helsinki (36). Informed consent was obtained from all participants prior to their participation in the study. Preregistration was also completed prior to the study and can be found here (https://osf.io/bqk5u).

Procedures were completed in a fixed order: a short health questionnaire, the HK-OxVPS, and a logMAR visual acuity test. The health questionnaire was a twelve-item questionnaire covering age, education, handedness, neurological condition, psychological condition, and current and past eye conditions. All data collection was completed in person in Hong Kong. The researcher visited the participant at their home, or at a community venue such as a church, cafe, park or soup kitchen. Participants received verbal standardized instructions prior to the test. Before beginning the assessment, participants were asked to confirm that their environment was comfortable with ample lighting, taking into account varying preferences. Participants who wore glasses used their regular corrective lenses throughout the entire session. The tests were administered by a researcher in a consistent format using the same prompts and materials across all locations and sessions. The researcher ensured that the tests were administrated in a quiet location with minimal distractions and support from family members. Although the variability in lighting conditions and testing environments did not create perfect test conditions, our approach more accurately reflects a real testing situation in which HK-OxVPS could be used in the future.

The original OxVPS is described in detail by Vancleef et al. (20). Any deviations from the original version are reported above. The resulting HK-OxVPS consisted of 10 short subtests. Following questioning the participant about any subjective complaints regarding their vision, a stimulus booklet was placed in front of the participant at the body midline. In the next six tasks simple images were shown and the participant was asked to either name the object, select the word that was most closely related to the object, pick two similar shapes, count the number of dots on the page, judge the orientation of a line, or pick two photographs of the same person. On the final page of the stimulus booklet, a short paragraph of text was presented, and the participant was asked to read it aloud. The experimenter kept their hand on the stimulus test booklet and turned the pages. In this way, they ensured a consistent pace throughout the test. The respondent was allowed to take as much time as they needed for each task; however, the reading task was timed. Words of instructions were repeated, and encouragement was given if needed. Participants were encouraged to answer even if they were unsure. Finally, participants completed two worksheets: a cancellation task and a figure copy task. Further details of the tasks can be found in Table 1 in Vancleef et al. (20). Through these ten tasks, HK-OxVPS aims to screen for a wide range of visual perceptual conditions: optic aphasia, associative agnosia, apperceptive agnosia, simultanagnosia, blindsight, Anton's syndrome, prosopagnosia, alexia, etc. (20).

To assess near visual acuity, participants held a Logarithmic Visual Acuity Card (37) positioned 40cm away from their eyes. They were then asked to read the English alphabetical letters on the card with both eyes open. Visual acuity scores were recorded as the row the participant could no longer read at least 50% of the letters (38).

## Results

### Description of the sample

Ninety-seven Cantonese-speaking participants took part in the study on normative data, but two did not meet the inclusion criteria and were excluded from the analyses. All remaining 95 participants were residents of Hong Kong and were of Chinese and Vietnamese Chinese ethnicity, of which 32 were male and 63 female. Participants' ages ranged from 30 to 95 years old (M= 68.54, SD= 11.62) and recruitment was stratified by age groups, so the distribution of ages would match the age distribution of Hong Kong stroke patients [(29), conform Guideline 9 and 11 in (22)]. Convenience sampling resulted in some age groups having slightly more or fewer participants than required, as seen in Table 1. Further details of all participants in the normative sample can be found in Table 2.

**Table 2:** Demographical details of participants in our normative sample.

	**Mean**	**SD**	**Count**	**Proportion**
Age (years)	68.54	11.62	95	
Gender				
Man			32	0.34
Woman			63	0.66
Education (years)	10.88	7.15	94	
Handedness				
Ambidextrous			3	0.03
Left			9	0.09
Right			83	0.88
Near visual acuity (logMAR)^*^	0.18	0.26	59	
Glasses				
Distance glasses			18	0.19
Reading glasses			8	0.08
Varifocal or bifocal glasses			16	0.17
None			52	0.55
Unsure			2	0.02
Eye condition^†^				
Glaucoma			3	0.03
Macular degeneration			5	0.05
Cataracts			25	0.26
Diabetic			1	0.01
Retinal detachment			1	0.01
Dry eye			2	0.02
Other			5	0.05
None			56	0.59
Unsure			3	0.03

^*^Thirty-six participants were unable to complete the near visual acuity test because they were unfamiliar with the English alphabet. Mean and SD reported here is from the remaining 59 participants.

^†^The total count data of “Eye condition” exceeds the total participant number of 95 because some participants had multiple eye conditions.

### Normative data and comparison with original OxVPS

Not all participants were able to complete all HK-OxVPS subtests. Across all participants there were 40 incomplete subtests (3.83%). Commonly incomplete subtests were Reading, Cancellation, and Figure Copy. Nine participants (9.47%) could not complete the Reading subtest due to being illiterate. In the Cancellation task, four participants (4.21%) struggled to understand instructions and could not complete the task. Moreover, seven participants (7.37%) could not complete the Figure Copy as they could not draw or hold a pen. Participants who could not complete all subtests were aged 56–95 (n = 18, M = 80.28, SD = 13.17) with years of education ranging from 0 to 19 years (M = 4.11, SD = 7.18). A substantial proportion of these participants reported having no formal education (66.67%) and were unable to complete the subtest due to illiteracy and functional writing difficulties.

Tables 3, 4 provide descriptive statistics for each of the HK-OxVPS subtests. Cut-off scores for each task were developed based on the 5th percentile of each subtest. Differences in cut-off scores suggest a performance discrepancy between the translation (HK-OxVPS) and the original version (OxVPS), with the HK-OxVPS having lower cutoff scores in nearly all subtests. However, it is essential to note that both versions of the reading task are in their respective language and are not suitable for direct comparison.

**Table 3:** Descriptive statistics for subtest 1–6.

**Subtest**	**OxVPS**				**HK-OxVPS**			
	***N***	**Median**	**IQR**	**P5th**	***N***	**Median**	**IQR**	**P5th**
1. Picture naming	107	4	4–4	3	95	4	4–4	2
2. Semantic information	107	4	4–4	3	95	4	3–4	2
3. Global shape perception	107	4	4–4	3	95	4	4–4	2
4. Item counting	107	4	4–4	4	95	4	4–4	2.7
5. Simple feature perception	107	4	4–4	4	95	4	4–4	2
6. Face recognition	107	4	4–4	2.3	95	4	3–4	2

All subtests here are scored out of 4 trials. *N* = number of participants who completed the subtest out of 95 participants (HK-OxVPS) and 107 participants (OxVPS). Descriptive statistics on OxVPS are taken from Vancleef et al. (20).

**Table 4:** Descriptive statistics for subtest 7–9.

**Subtest**	**OxVPS**				**HK-OxVPS**			
	***N***	**Median**	**IQR**	**P5th**	***N***	**Median**	**IQR**	**P5th**
7a. Reading (speed cpm)	105	138.5	122.1–150	93.5	79	193.6	172.6–220.9	76.8
7b. Reading (accuracy of irregular words)	105	10	10–10	9	86	31	31–31	29
8a. Cancellation (object)	107	0	0–0	0 and 0	91	0	0–0	0 and 0
8b. Cancellation (space)	107	0	0–0	−2 and 2	91	0	0–1	−2 and 2.75
9a. Figure copy (score)	107	60	59–60	56	88	60	59–60	48
9b. Figure copy (strategy)	51				88			

All subtests here are scored differently [see Vancleef et al. (20)]. *N* = number of participants who completed the respective subtest out of 95 participants (HK-OxVPS) and 107 participants (OxVPS). Statistics about OxVPS are taken from Vancleef et al. (20). Cpm, characters per minute.

Angoff's delta plot method (39) was used to evaluate comparativeness of both of the OxVPS, as recommended by the International Neuropsychological Society's guidelines for translating and adapting tests [Guideline 10 in (22)]. This method compares the proportions of correct responses between the HK-OxVPS and OxVPS through a statistical transformation of item difficulty on a standardized scale of each item: proportions correct are first transformed to Z-scores and then to delta values (delta = 4^*^Z+13) (40). The higher the delta value the more difficult the item. The difficulty of each item in the HK-OxVPS is compared with the difficulty of its counterpart in OxVPS. Subtests 7–10 (see Table 4, subtest 10 is a self-evaluation of a participant's vision) were not included because the analysis requires a dichotomous dependent variable: individual items that can be scored as correct or incorrect rather than a sum score. Although subtests 7–10 technically have individual items (each heart, line, or character) their meaning and correctness depends on the other items in the subtest and the assumption of independence of items is therefore violated. The R deltaPlotR package (40) was used to run this analysis with the HK-OxVPS scores set as the focal group.

**Figure 2:**
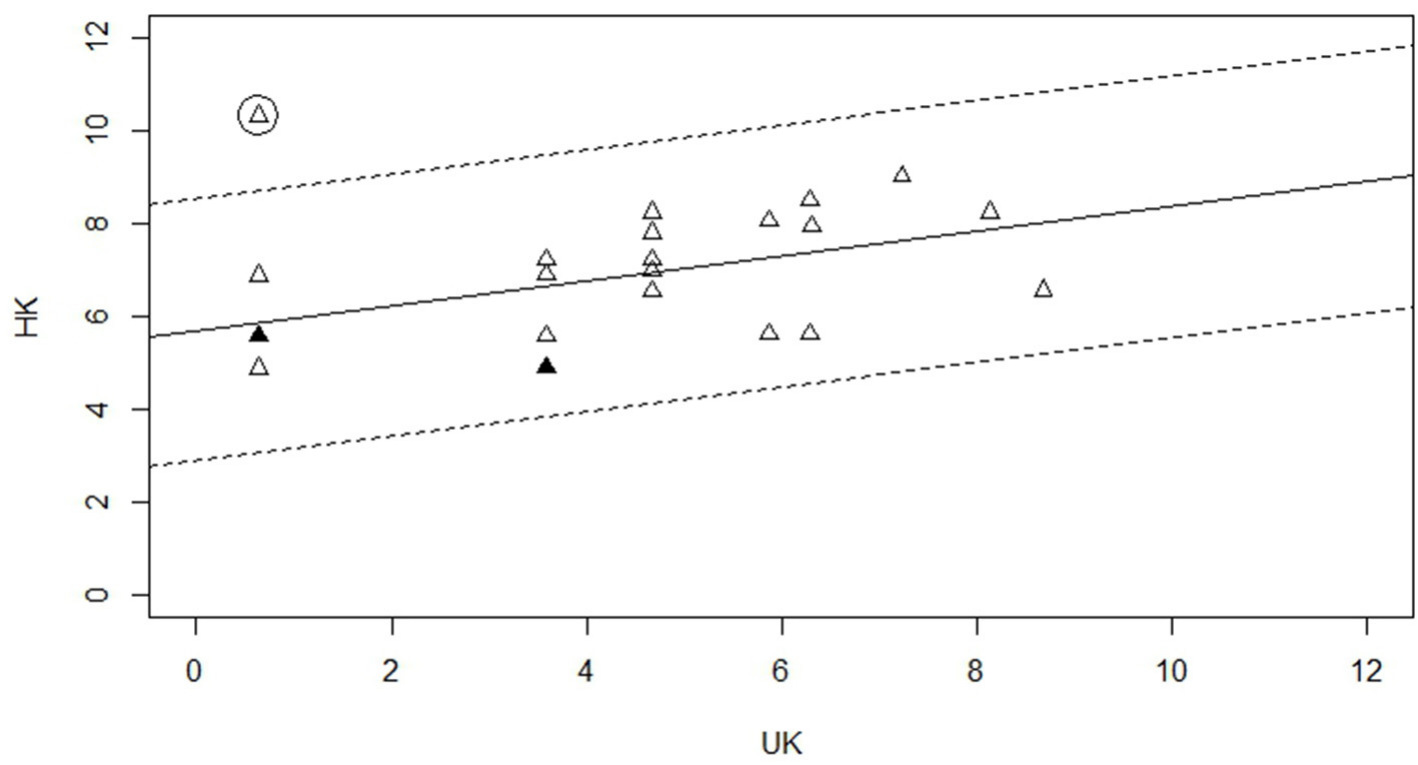
Delta Plot showing the delta values for the HK-OxVPS and OxVPS items of subtest 1–6. The filled in triangle indicates more than one point and the circled triangle indicates a significant DIF item.

The delta plot analysis estimated the parameters of the major axis at 5.69 for the intercept *a* and 0.27 for the slope *b* and established an appropriate DIF classification threshold at 2.73 (significance level: 5%). Figure 2 shows a scatterplot of delta points with the horizontal axis showing the delta values of the 24 OxVPS items of subtest 1–6 and the vertical axis showing the delta values of the corresponding 24 HK-OxVPS items. The line of best fit in Figure 2 represents the expected relationship of item difficulty uniformity across both versions and is the major axis. We observed a positive intercept (*a* = 5.69) suggesting that the items in the HK-OxVPS are generally harder than the original OxVPS. This can also be seen in Figure 2: the major axis is shifted upwards from the identity line and the delta points in the left half of the plot show higher delta values for the HK-OxVPS compared to the original OxVPS indicating that these items are substantially harder in the HK-OxVPS than in the original OxVPS. The small slope of 0.27 indicates that both versions of OxVPS show varying levels of difficulty. In other words, there is only a weak relationship between item difficulty in both versions. In Figure 2, this can be seen in the clockwise tilt of the major axis. Taken together, this pattern suggests potentially differential item functioning (DIF). Further analysis with a DIF classification threshold, adjusted to the distribution, of the delta values (2.73 at significance level of 0.05) showed that item 6 (semantic information question 2) was flagged as DIF (indicated with a circle in Figure 2).

### Exploring subtest specific effects of age, education, and visual acuity

A multivariate analysis of covariance (MANCOVA) was conducted to explore the effect of participant demographic characteristics: age, education, and visual acuity, on test performance in all subtests. For this analysis, data of 57 participants was available: for 36 participants we did not have a measure of visual acuity, and two more participants could not complete the reading task. We observed high correlations between predictor variables age and education (*n* = 57, *r* = −0.45, *p* < 0.001) and between acuity and age (*n* = 57, *r* = 0.23, *p* = 0.09), but not between acuity and education (*n* = 57, *r* = −0.08, *p* = 0.54). We centered all variables on their mean value to reduce the impact of this multicollinearity, and we used Type III sum of squares. In addition, all 57 participants scored 0 on the object-based neglect score in the cancellation task. The lack of variance meant this variable was excluded from the analysis.

The MANCOVA indicated no statistically significant effect of age [Pillai's trace = 0.07, *F*_(10, 44)_ = 0, *p* = 0.96] or education [Pillai's trace = 0.26, *F*_(10, 44)_ = 2, *p* = 0.16], and a borderline significant effect of visual acuity [Pillai's trace = 0.31, *F*_(10, 44)_ = 2, *p* = 0.07]. The effects of education and age were not further explored per subtest of HK-OxVPS with univariate analyses of variance (ANOVA), but the effect of visual acuity was. The detailed results are presented in Table 5: the effect of visual acuity was most prominent in the Simple Feature Perception task.

**Table 5:** ANOVA results for the effect of visual acuity.

**Subtests**	***F*_(10, 44)_**	***p***
1. Picture naming	3.68	0.06
2. Semantic information	0.46	0.50
3. Global shape perception	1.68	0.20
4. Item counting	0.18	0.67
5. Simple feature perception	15.21	<0.001
6. Face recognition	0.12	0.73
7a. Reading (speed cpm)	2.25	0.14
7b. Reading (accuracy irregular words)	0.39	0.53
8b. Cancellation (space)	0.57	0.45
9a. Figure copy (score)	3.44	0.69

Cpm, characters per minute.

However, it is important to highlight that only 57 participants (60%) were included in the MANCOVA. Thirty-eight participants were unable to complete the reading or visual acuity task because they were unfamiliar with the English alphabet or illiterate. Participants who completed all tests (mean age = 64) were on average younger than the participants with missing data [mean age = 75, *t*_(60)_ = −4.49, *p* < 0.001] and had completed more years of education [13 years vs. 7 years, *t*_(64)_ = 4.66, *p* < 0.001]. Exclusion of older and lower educated participants might have biased results and careful interpretation of the MANCOVA results is recommended.

## Discussion

This study aimed to develop a cultural adaptation and translation of the OxVPS, the HK-OxVPS, for screening for visual perception difficulties among Cantonese-speaking stroke survivors in Hong Kong, with potential application across East Asia, and in immigrant communities worldwide. Normative data was collected from the Cantonese-speaking Hong Kong middle to old-aged population, and scores were compared with those from the original version of the OxVPS. Further exploratory investigations on the effect of age, education, and visual acuity on test performance and completion were carried out.

### Equivalence of test versions

Overall, results from this study show overall reduced scores in the HK-OxVPS compared to the original OxVPS. This subsequently also affected cutoff scores for each respective subtest. In addition, a higher percentage of participants were unable to complete the HK-OxVPS reading task (9.74%) compared to the OxVPS reading task (1.87%).

A first potential explanation for the differences in scores might be that the cultural adaptations have been insufficient. Stimuli that were deemed culturally unfamiliar for the Cantonese population in the picture naming, semantic information, and facial recognition tasks, were replaced in the HK-OxVPS by culturally relevant alternatives. For the reading task, a traditional Chinese passage was written. Despite piloting these changes with a small group of Cantonese speaking participants, we still observed an unintended difference in difficultly level between both versions. Potentially, our younger, more educated group of pilot participants were more with a Western culture than our normative sample. Therefore, cultural appropriateness for a general population might have been overestimated. If so, further cultural adaptations might bring the normative HK-OxVPS scores more in line with the scores of the normative sample of the original OxVPS.

Secondly, the difference in educational levels of the OxVPS and HK-OxVPS samples might have been at the underlying basis of the discrepancy in scores. The normative sample in the original OxVPS includes mostly highly educated participants with 76% completing post-secondary education, equivalent to 14–20 years of education. In contrast, our HK-OXVPS normative sample had an average of 10.33 years of education. This is likely more representative of Hong Kong society where only 17% of over 50-year-olds have completed post-secondary education (41). Many of Hong Kong's ageing population are refugees or immigrants from China during the Maoist era in China. During events such as the Great Leap Forward, the Great Chinese Famine and the Cultural Revolution, Hong Kong accepted a sizeable and continuous influx of Chinese immigrants seeking refuge in what was then still a British colony (42). By the 1960s, a third of Hong Kong's population was from China (43). While Hong Kong had adopted the British education system with an emphasis on free and compulsory education, those from China were not able to receive any formal education. This demographic of immigrants from China now makes up a large percentage of the elderly population. Having received minimal to no education in the early 1960s, many of them are still unable to read or write and as a result unable to complete any reading- or literacy-related tests (44). Therefore, the difference in scores between the original OxVPS and the HK-OxVPS could reflect a difference in education between both samples rather than a cultural difference (see below for a further discussion on the education). If so, the normative data and cut-off scores of the original OxVPS might not be appropriate for lower educated people, even within the United Kingdom.

**Figure 3:**
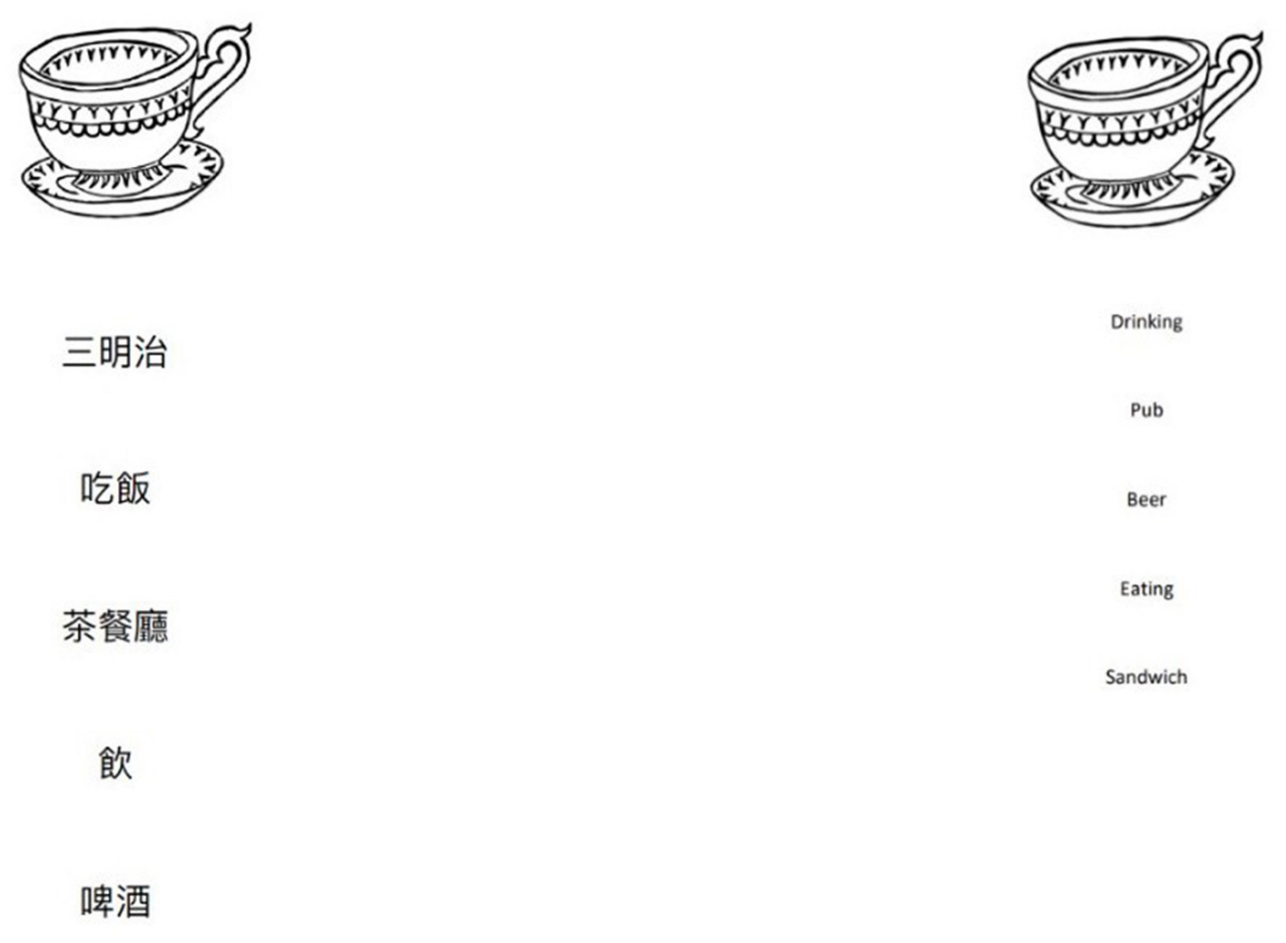
Image of item 6 for both versions. The Item 6 HK-OxVPS answer options are translated as follows: “*saam man zi”* sandwich, “*sik fan”* to eat, “*cha chaan teng”* cantonese-style cafe, “*yum”* drink, “*be zau”* beer. © University of Durham.

One semantic information item (Item 6, Figure 3) was flagged for DIF, indicating a disproportionately large discrepancy in performance between the HK-OxVPS and the OxVPS versions, even after controlling for overall increased difficulty in HK-OxVPS. This stimulus for both versions is identical (see Figure 3) and familiar to a Hong Kong population, with the correct semantic answer being “drink.” Within the HK-OxVPS the answer options “pub” was replaced with 茶餐廳 (“cha chaan teng”), a Cantonese-style café. This change may explain why a certain number of participants chose “Cantonese-style café” instead of “drink,” as a similar type of cup could potentially be used in such establishments. Future iterations of the HK-OxVPS could modify this answer option to 酒吧 (“zou ba”), which translates to “bar” and is more similar to the answer option “pub” in the OxVPS.

The discrepancies in scores between the two versions highlight the issue of the blind application of translated Western tests in non-Western populations using the same normative cutoff scores, even for visual perception tests. Many have used discrepancies in scores on neuropsychological tests to make the false claim that certain ethnic groups possess lower intelligence due to their lower scores with the justification that these tests are “culture-fair” as they can be completed non-verbally (56). Whereas, research showed that perception, manipulation, and conceptualization of visuo-spatial information vary considerably across different cultures (45). The findings from our study confirm that visual perception tests are indeed not culture-free. This highlights the need to develop culturally appropriate versions of neuropsychological tests including visual perceptual tests with culture-specific normative data. As Nguyen et al. (22) recommend (Guideline 12), scores should be interpreted with reference to appropriate populations norms collected from the same cultural group and comparing raw scores across populations should be avoided (Guideline 16). Therefore, the discrepancy in scores between OxVPS and HK-OxVPS is not necessarily preventing the use of HK-OxVPS in a Hong Kong population as long as appropriate normative data is used and the HK-OxVPS scores are not compared with scores on the original OxVPS. The goal of our cultural adaptation was indeed not to produce OxVPS versions with equivalent scores, but versions that measure equivalent constructs.

### Effect of age, education, and visual acuity on performance and completion

The results of the analysis on the effect of age, education, and visual acuity on performance on the HK-OxVPS need to be interpreted with care. Despite the fact that an English letter chart is the most common eye chart in adult primary care in Hong Kong (46), 36 participants in our study were unable to complete the LogMar visual acuity test due to being unfamiliar with the English alphabet. They could therefore not be included in our MANCOVA. Our pilot study did not highlight the reading chart as an issue, likely due to an underrepresentation of lower educated participants in our pilot sample. Research on visual acuity testing in Hong Kong has highlighted challenges unique to the region. According to Van Newkirk (46), a significant barrier to completing visual acuity assessments is the inability of some individuals to recognize English alphabetical letters. This issue is particularly relevant given Hong Kong's generational education patterns shaped by its colonial history, where English proficiency has varied across age groups and educational backgrounds (47). Future research should explore alternative visual acuity and visual perception assessment methods that accommodate these linguistic barriers. A culturally appropriate test could improve completion rates and ensure more accurate evaluations for individuals with limited English literacy. Until then, our finding of an absent effect of visual acuity, education, and age should be interpreted with caution as the analysis might have been biased toward people with higher education and good familiarity with the English alphabet. Future studies should investigate the effects of visual acuity, age, and education in a more representative sample for whom visual acuity is measured with symbols or through matching.

The absence of effect of age on the HK-OxVPS in our sample is in contrast to prior studies on the interaction of visual perception abilities with age, where older adults of comparable age to our sample exhibit decreased visual sensory activation during visual perceptual tasks (48–50). Other Hong Kong-related screening and diagnostic tests, such as the Oxford Cognitive Screen—Putonghua (51) also showed patterns of cognitive and visual-related declines and have opted to adapt age-specific cutoff scores for better representation and age-specific generalisability (52). Visual acuity was only found to affect performance on Simple Feature Perception task of the HK-OxVPS. These results contrast earlier findings of an effect of visual acuity on Global Shape Perception and Figure Copy scores in Vancleef et al. (20). Similarly, education did not affect HK-OxVPS performance scores in our sample of mostly highly educated older volunteers. This was also seen in the Hong Kong version of the Oxford Cognitive Screen (HK-OCS) (52). In the Italian and Dutch version of the Oxford Cognitive Screen, education effects were limited to the number of crossed out items in the cancellation task (53, 54). Like our MANCOVA sample, their sample included participants with, on average, 14 years of education (52, 53). In a sample with on average 8.6 years of education, education did have an effect on two out of eight subtests of the Visual Object and Space Perception battery (50). This pattern of results across studies might suggest that education can affect scores on neuropsychological assessments for visual perception in people who did not complete secondary education but has less of an effect in highly-educated people.

### Directions for future research

As convenience sampling was used during participant recruitment, the potential for sampling bias and volunteer bias raises the concern of unrepresentativeness of the Hong Kong middle- to old-age population. An attempt to minimize this bias was made by recruiting in various locations including soup kitchens typically attended by people from lower socio-economic backgrounds. The range in the participants' educational background testifies to the success of this recruitment strategy. For future studies, we would recommend recruitment in such locations, in addition to traditional recruitment locations to include participants with lower education.

Nguyen et al. (22) recommend intensively reviewing test procedures and answer options in and with the target population (Guideline 3, 7, and 13). Because of limited resources we were unable to check whether the book format was appropriate for the population, whether encouraging people to guess was culturally appropriate, and whether people in Hong Kong were impacted by the administrator recording the duration of the cancellation and reading task. We would recommend researchers adapt tests for other cultural contexts to thoroughly pilot administration of the test and to conduct more in-depth interviews with pilot participants regarding their answer strategy. A thinking-a-loud interview method might be useful in this context. We did not specifically evaluate the impact of testing conditions such as teleneuropsychology, bed-bound patients, patients with communication difficulties or patients with motor impairments. This is because the design of the original OxVPS already considered inclusivity for such conditions that would similarly impact a Hong Kond patient population. [Guideline 14 in (22)].

While this study reports normative data and describes cut-off score for the HK-OxVPS as well as the relationships with demographic variables [conform Guideline 17 in (22)], a translation of the user manual [Guideline 18 in (22)], and further validity and reliability testing in patient populations should be completed. Convergent validity and discriminant validity can be tested by correlating with other established visual perception tests (convergent validity) and with unrelated assessment measures such as with verbal memory or executive function tests (discriminant validity). Reliability research could also help ensure screening test results are consistent and stable. This can be done by administering the same measure across different time points to evaluate test-retest reliability or have multiple raters assess for agreement for inter-rater reliability (55). Validity and reliability testing should be prioritized in additional research for further establishment of credibility and usability for clinical usage in Hong Kong for visual perception screening.

## Conclusion

Our results highlight how crucial it is to modify assessments to the cultural and language background of the population in order to provide fair and accurate judgements. Assessment techniques that do not take into account cultural and language variations may result in incorrect diagnoses, misinterpretations of patients' needs, and disparities in the provision of healthcare. Despite the limitations highlighted above, we would argue that, except for item 6, our cultural adaptation and translation of the OxVPS, the HK-OxVPS, can be used in a Hong Kong population as long as the Hong Kong normative data and cut-off scores are used. However, we would advise against using the Reading task in this population unless pre-stroke literacy is confirmed [conform Guideline 15 in ref. (22)]. Within Hong Kong's clinical setting, the HK-OxVPS could provide practical value. The knowledge gained from this study not only broadens understanding of the need for culturally sensitive assessments but also provides healthcare professionals in Hong Kong with specific advice, highlighting the need for patient-centered treatment.

## Data Availability

The datasets presented in this study can be found in online repositories. The names of the repository/repositories and accession number(s) can be found below: https://osf.io/v2hgz/.
